# Relationship between preoperative statins use and atrial fibrillation (AF) after cardiac surgery. analysis with a propensity score

**DOI:** 10.1186/2197-425X-3-S1-A948

**Published:** 2015-10-01

**Authors:** M Fernandez-Zamora, A Gordillo-Brenes, MD Arias-Verdú, E Curiel-Balsera, A Herruzo-Aviles, M Garcia-Delgado, JA Arboleda-Sánchez, A Hinojosa-Perez, R Rivera-Fernández

**Affiliations:** Intensive Care Unit, Hospital Regional Universitario Carlos Haya, Málaga, Spain; Intensive Care Unit, Hospital Puerta Del Mar, Cádiz, Spain; Intensive Care Unit, Hospital Virgen del Rocio, Sevilla, Spain; Hospital Virgen de las Nieves, Granada, Spain; Intensive Care Unit, Hospital Regional Universitario Carlos Haya, Spain, Spain; Intensive Care Unit, Hopspital Virgen del Rocio, Sevilla, Spain

## Objectives

To analyze the relationship between preoperative statin use and the development of atrial fibrillation (AF) in postoperative cardiac surgery patients.

## Methods

Prospective cohort study of cardiac surgery patients in 11 hospitals , included in the Spanish ARIAM register, during 2008-2012. Users of statins before surgery were matched to non-users according to a propensity score to quantify the probability of being treated with statins preoperatively, based on demographics, comorbidities, medication and type of surgery. We analyzed differences in the incidence of postoperative atrial fibrillation in both groups. The statistical study was done with the Student T, X^2,^ Mc-Nemar test, and logistic regression.

## Results

N= 7,276 patients. Mean age: 63.91 ± 12 45 years. Elective surgery: 85.9%. Surgical risk EuroSCORE: 5.86 ± 3.14 points. ICU mortality: 7.6%.Hospital mortality 10.1% (8.1% missing).Before surgery, 51.5% of patients had taken statins and 25.8 % had atrial fibrillation. After intervention, 21.9% presented atrial fibrillation as a complication during ICU stay.Among patients who were treated with statins preoperatively, AF occurred in 19.8% vs 22.5% in those no taking (P = 0.006). By multivariate analysis with logistic regression was observed that the frequency of AF as a complication to equal the other variables included in the model (prior history of AF, EuroSCORE, SAPS-3, urgent surgery and surgical valve type , time By-pass more than 120 minutes) was not lower in patients taking statins preoperatively (OR: 0.975, CI: 0.861-1.105). After, we performed the analysis in the group of patients matched by propensity index to receive preoperatorive statins (1528 treated with statins and 1528 without statins) and we observed that the frequency of FA was 20.4% in patients treated with statins (1528) and 24.9 % in 1528 untreated (p = 0.003), OR: 0.772, CI: 0652-0916).Logistic regression performed in the group of patients matched by propensity score to receive preoperative statins (1528 treated with statins and 1528 without statins) was observed that the frequency of AF to equal the other variables included in the model (prior history of AF, SAPS-3, urgent surgery and surgical valve type, bypas time more than 120 minutes) was lower in patients taking statins preoperatively (OR: 0.779, CI: 0649-0936).

## Conclusions

Preoperative statin use was associated with a lower risk of postoperative atrial fibrillation after cardiac surgery.

